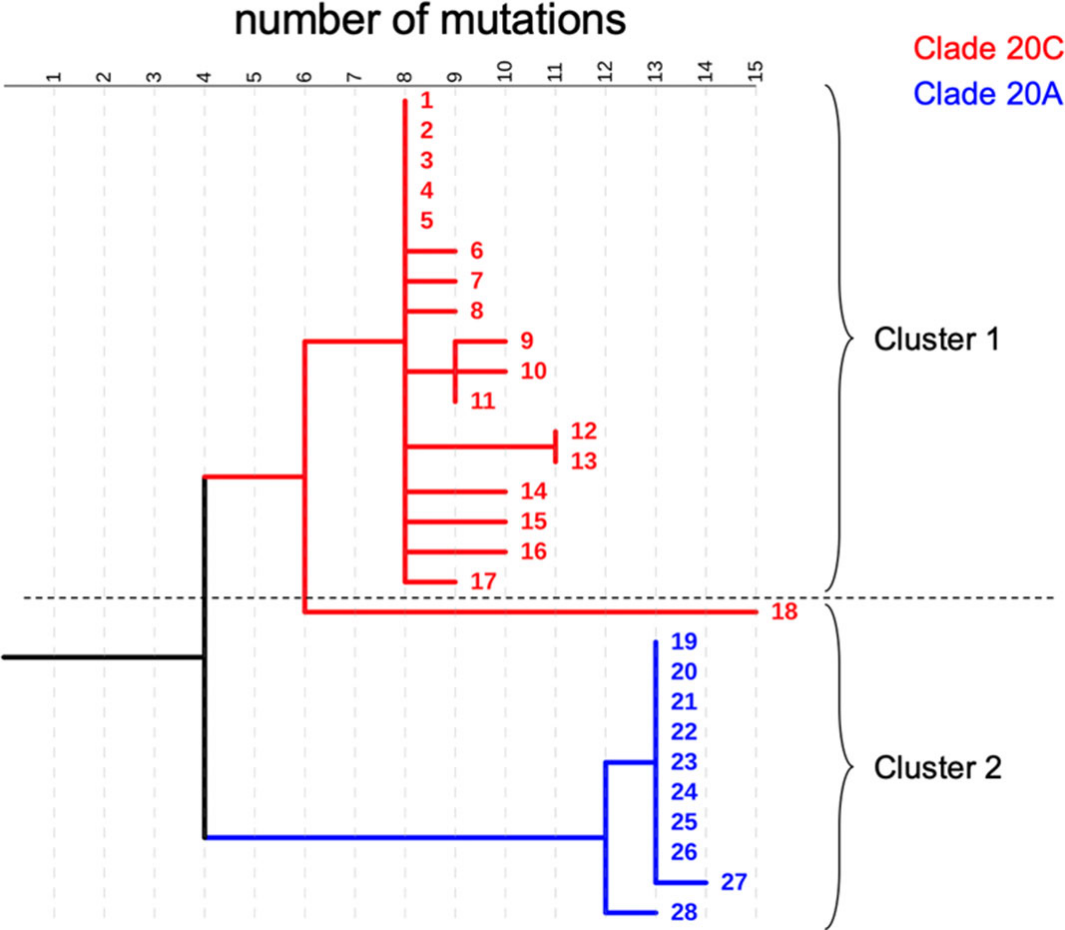# Molecular Epidemiology of Large COVID-19 Clusters at an Academic Medical Center, March–October 2020

**DOI:** 10.1017/ash.2021.19

**Published:** 2021-07-29

**Authors:** Takaaki Kobayashi, Miguel Ortiz, Stephanie Holley, William Etienne, Kyle Jenn, Oluchi Abosi, Holly Meacham, Lorinda Sheeler, Angie Dains, Mary Kukla, Alexandra Trannel, Alexandre Marra, Mohammed Alsuhaibani, Paul McCray, Stanley Perlman, Bradley Ford, Daniel Diekema, Melanie Wellington, Alejandro Pezzulo, Jorge Salinas

## Abstract

**Background:** COVID-19 in hospitalized patients may be the result of community acquisition or in-hospital transmission. Molecular epidemiology can help confirm hospital COVID-19 transmission and outbreaks. We describe large COVID-19 clusters identified in our hospital and apply molecular epidemiology to confirm outbreaks. **Methods:** The University of Iowa Hospitals and Clinics is an 811-bed academic medical center. We identified large clusters involving patients with hospital onset COVID-19 detected during March–October 2020. Large clusters included ≥10 individuals (patients, visitors, or HCWs) with a laboratory confirmed COVID-19 diagnosis (RT-PCR) and an epidemiologic link. Epidemiologic links were defined as hospitalization, work, or visiting in the same unit during the incubation or infectious period for the index case. Hospital onset was defined as a COVID-19 diagnosis ≥14 days from admission date. Admission screening has been conducted since May 2020 and serial testing (every 5 days) since July 2020. Nasopharyngeal swab specimens were retrieved for viral whole-genome sequencing (WGS). Cluster patients with a pairwise difference in ≤5 mutations were considered part of an outbreak. WGS was performed using Oxford Nanopore Technology and protocols from the ARTIC network. **Results:** We identified 2 large clusters involving patients with hospital-onset COVID-19. Cluster 1: 2 hospital-onset cases were identified in a medical-surgical unit in June 2020. Source and contact tracing revealed 4 additional patients, 1 visitor, and 13 employees with COVID-19. Median age for patients was 62 (range, 38–79), and all were male. In total, 17 samples (6 patients, 1 visitor, and 10 HCWs) were available for WGS. Cluster 2: A hospital-onset case was identified via serial testing in a non–COVID-19 intensive care unit in September 2020. Source investigation, contact tracing, and serial testing revealed 3 additional patients, and 8 HCWs. One HCW also had a community exposure. Patient median age was 60 years (range, 48–68) and all were male. In total, 11 samples (4 patients and 7 HCWs) were sequenced. Using WGS, cluster 1 was confirmed to be an outbreak: WGS showed 0–5 mutations in between samples. Cluster 2 was also an outbreak: WGS showed less diversity (0–3 mutations) and ruled out the HCW with a community exposure (20 mutations of difference). **Conclusion:** Whole-genome sequencing confirmed the outbreaks identified using classic epidemiologic methods. Serial testing allowed for early outbreak detection. Early outbreak detection and implementation of control measures may decrease outbreak size and genetic diversity.

**Funding:** No

**Disclosures:** None

**Figure 1.**